# A Highly Active Chimeric Lysin with a Calcium-Enhanced Bactericidal Activity against *Staphylococcus aureus* In Vitro and In Vivo

**DOI:** 10.3390/antibiotics10040461

**Published:** 2021-04-19

**Authors:** Xiaohong Li, Shujuan Wang, Raphael Nyaruaba, Huan Liu, Hang Yang, Hongping Wei

**Affiliations:** 1 CAS Key Laboratory of Emerging Pathogens and Biosafety, Centre for Biosafety Mega-Sciences, Wuhan Institute of Virology, Chinese Academy of Sciences, Wuhan 430071, China; lixiaohong@wh.iov.cn (X.L.); 13429827792@163.com (S.W.); rohuru1@gmail.com (R.N.); HuanL88xiao@163.com (H.L.); 2 College of Life Sciences, University of Chinese Academy of Sciences, Beijing 100049, China; 3 Sino-Africa Joint Research Center, Chinese Academy of Sciences, Wuhan 430074, China

**Keywords:** *Staphylococcus aureus*, lysin, bactericidal activity, calcium, bacteremia, ClyC

## Abstract

Lysins, including chimeric lysins, have recently been explored as novel promising alternatives to failing antibiotics in treating multi-drug resistant (MDR) pathogens, including methicillin-resistant *Staphylococcus aureus* (MRSA). Herein, by fusing the CHAP (cysteine, histidine-dependent amidohydrolase/peptidase) catalytic domain from the Ply187 lysin with the non-SH3b cell-wall binding domain from the LysSA97 lysin, a new chimeric lysin ClyC was constructed with Ca^2+^-enhanced bactericidal activity against all *S. aureus* strains tested, including MRSA. Notably, treating *S. aureus* with 50 μg/mL of ClyC in the presence of 100 μM Ca^2+^ lead to a reduction of 9 Log_10_ (CFU/mL) in viable bacterial number, which was the first time to observe a lysin showing such a high activity. In addition, the effective concentration of ClyC could be decreased dramatically from 12 to 1 μg/mL by combination with 0.3 μg/mL of penicillin G. In a mouse model of *S. aureus* bacteremia, a single intraperitoneal administration of 0.1 mg/mouse of ClyC significantly improved the survival rates and reduced 2 Log_10_ (CFU/mL) of the bacterial burdens in the organs of the infected mice. ClyC was also found stable after lyophilization without cryoprotectants. Based on the above observations, ClyC could be a promising candidate against *S. aureus* infections.

## 1. Introduction

*Staphylococcus aureus* is a common Gram-positive pathogen that colonizes the human surface skin [1,2]. This opportunistic pathogen can cause severe infections, such as pneumonia, endocarditis, osteomyelitis, meningitis, and bacteremia [3]. *S. aureus*-related infections have become a challenge due to the emergence of isolates that are resistant to different antibiotics including methicillin (MRSA) [4]. To combat this threat, novel antimicrobial alternatives with different mechanisms of action from conventional antibiotics are urgently needed [5,6].

Endolysins (or lysins) are hydrolytic enzymes capable of specifically recognizing target bacterial cell wall and cleaving its peptidoglycan for the release of progeny virions during the lytic cycle of phages [7,8]. This specificity, coupled with the low resistance risk of lysins, have made them promising alternatives to failing antibiotics in combating multi-drug resistant (MDR) pathogens [9,10]. Some phage lysins have been reported to have good activities against *S. aureus* and are in clinical trials now [11,12,13]. However, some lysins may have dose-dependent toxicity [14]. It is important for a lysin drug candidate to possess as high as possible activity in order to have a better therapeutic index.

Utilizing the modular structure property of phage lysins, i.e., one or two catalytic domains (CDs) in the *N*-terminus and one cell-wall binding domain (CBD) in the C-terminus, chimeric lysins have been constructed to improve lysis activity [15], extend bactericidal spectrum [16], or improve stability [17], etc. For example, Yang et al. constructed a chimeric lysin, ClyR, by fusing the CHAP (cysteine, histidine-dependent amidohydrolase/peptidase) domain from the PlyC lysin with the CBD from the PlySs2 lysin [16,18]. This combination resulted to a newly reported chimeric lysin with robust bactericidal activity and an extended-spectrum streptococcal host range that includes multiple streptococcal species, as well as representative species of enterococci and staphylococci (including MRSA and VISA). Apart from chimeric lysins, other techniques like fusion of natural lysins or parts of their domain with components of proteins or peptides (artilysins) [19] also exist on engineering of lysins. These techniques can be summarized into first generation lysins (wild type lysins as antimicrobial agents), second generation lysins (engineered to improve antibacterial and biochemical properties), and third generation lysins (engineered to improve pharmacokinetics and/or pharmacodynamics) [20].

There are also reports that the activity of some lysins could be enhanced by host factors in serum [21]. CF-301 showed substantially greater potency (32 to 100-fold) in human blood versus the normal buffers by acting synergistically with two key human blood factors, human serum lysozyme (HuLYZ) and human serum albumin (HSA). This finding may have important therapeutic implications for durable improvements in clinical outcomes of serious antibiotic-resistant staphylococcal infections.

In this study, we aimed to find chimeric lysins with high activity, which could be enhanced further by metal ions, especially Ca^2+^, in human blood. Ca^2+^ is suitable because calcium ion is one of the abundant metallic ions necessary in the human body. The free calcium ion in the human blood reaches up to 1200 mM [22]. It has also been found that some natural lysins contain domains capable of binding Ca^2+^ and their activity depends on Ca^2+^ [23,24]. We formed a new chimeric lysin, ClyC, by fusing the CHAP catalytic domain from the Ply187 lysin [25,26] with the non-SH3b CBD from the LysSA97 lysin [27]. Ply187 is a *S. aureus* bacteriophage 187 endolysin that was initially reported by Loessner et al. [25]. This lysin is unique among Gram-positive lysins because it lacks a CBD [15]. To improve its activity, the CHAP domain of Ply187 has been fused with the CBD of other lysins to form a new chimera. Similarly, we fused the CHAP domain of Ply187 with the non-SH3b CBD of LysSA97 to form the new chimera, ClyC. The non-SH3b CBD used is derived from the bacteriophage SA97 endolysin, LysSA97 [27,28]. Among *S. aureus* lysins, it is not common to find lysins without the SH3 domain. In a study by Chang and Ryu [28], after analysis of 98 endolysin genes of *S. aureus* phages, it was found that most lysins have a known CBD (80.61%) with a src-homology 3 (SH3, PF08460) domain, while few lysins (19.39%) like LySA97 have a novel putative CBD at their C-terminal.

The formed chimeric lysin, ClyC, showed a reduction of 9 log_10_ (CFU/mL) in viable bacterial number in the presence of Ca^2+^. More interesting, ClyC was found stable after lyophilization without stabilizing excipients. These properties indicated that ClyC could be a good candidate for further development into a drug for the treatment of infections caused by *S. aureus*.

## 2. Results

### 2.1. Construction and Purification of ClyC

**Figure 1:**
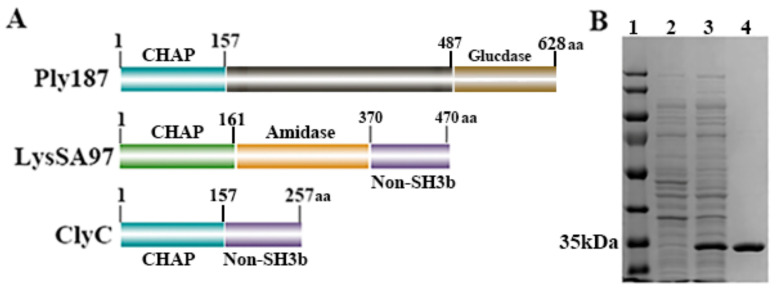
Construction of chimeric lysin ClyC. (**A**) Schematic representation of ClyC. (**B**) SDS-PAGE analysis of ClyC. Lane 1: Standard protein marker. Lane 2: Total bacterial solution before induction. Lane 3: Total bacterial solution after induction. Lane 4: Purified protein.

As shown in Figure 1A, a new chimeric lysin, ClyC (257 amino acids), was constructed by fusing the *N*-terminal CHAP domain of the Ply187 lysin (157 amino acids) with the C-terminal non-SH3b binding domain of the LysSA97 lysin (100 amino acids). SDS-PAGE analysis showed that ClyC could be expressed as a soluble protein in *E. coli* and the purified ClyC protein displayed high purity (>90%) (Figure 1B).

### 2.2. Bactericidal Activity of ClyC

**Figure 2:**
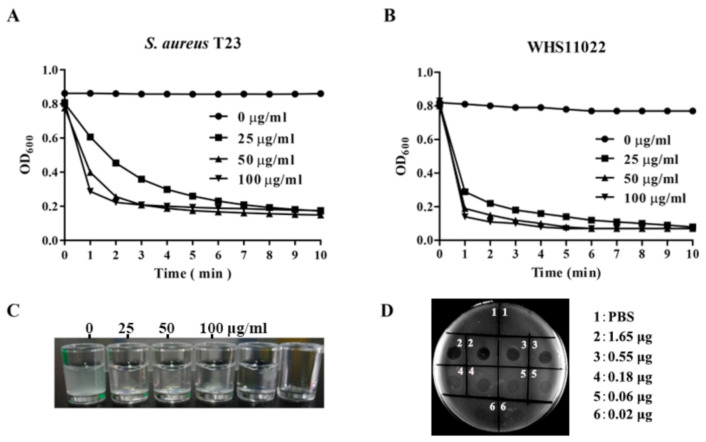
Bactericidal activity of ClyC. Lytic activity of ClyC against MRSA T23 (**A**) and *S. aureus* WHS11022 (**B**) at different concentrations (0, 25, 50, and 100 μg/mL). (**C**) Representative pictures of *S. aureus* T23 suspensions after treatment with different concentrations (0, 25, 50, and 100 μg/mL) of ClyC for 10 min. (**D**) Plaques assay result showing ClyC (0.02–1.65 μg/area) activity against *S. aureus* T23.

The bactericidal activity of ClyC was determined using the plaque assay and turbidity experiments. In the turbidity reduction experiment, the OD_600_ values of two representative *S. aureus* bacterial suspensions reduced rapidly within 1 min after exposure to ClyC (Figure 2A,B), showing similar slopes in the time-killing curves. The bacterial suspension also became clear after treatment with 25 μg/mL of ClyC for 10 min (Figure 2C), which meant that *S. aureus* cells were lysed quickly by ClyC. In support, clear plaques could also be seen in the plate after dipping 0.02–1.65 μg of ClyC on the lawn of *S. aureus* (Figure 2D). These results collectively demonstrated that ClyC is active against *S. aureus.* Further, the lytic spectrum of ClyC was determined with multiple bacterial strains. As shown in Figure S1, all 32 *S. aureus* strains and 6 other staphylococcal isolates tested were susceptible to ClyC. Notably, the lytic activity of ClyC was almost the same among all *S. aureus* strains tested within 10 min (Figure S1). However, ClyC was not active against streptococcus, enterococcus, and listeria. In addition, the MICs of ClyC against multiple *S. aureus* isolates were further determined. As shown in Table S3, ClyC showed MIC’s of 18 μg/mL for 19 isolates, 9 μg/mL for 7 isolates, 36 μg/mL for 2 isolates, and 4.5 μg/mL for 1 isolate, resulting in an overall average MIC of 16.6 μg/mL (SD ± 6.9).

### 2.3. Characteristics of ClyC

**Figure 3:**
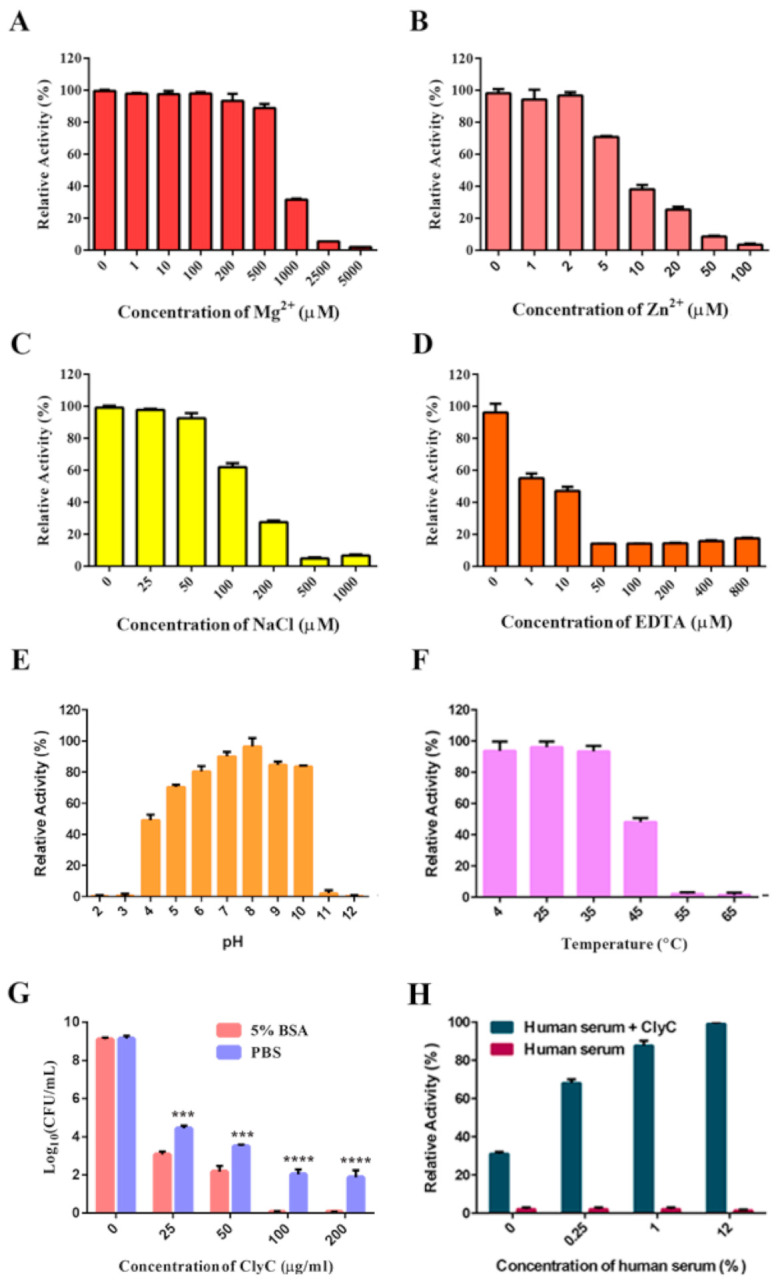
Physicochemical properties of ClyC. Effects of Mg^2+^ (**A**), Zn^2+^ (**B**), NaCl (**C**), EDTA (**D**), pH (**E**), temperature (**F**), PBS containing 5% BSA (**G**), and PBS containing human serum (**H**) on the antibacterial activity of ClyC. The relative activities of ClyC (25 μg/mL) were obtained by detecting the change of OD_600_ value against *S. aureus* T23 under different conditions. Log killing activities of different concentrations of ClyC (0, 25, 50, 100, and 200 μg/mL) against *S. aureus* T23 were determined in PBS with and without 5% BSA. All experiments were repeated three times. Error bars represent standard deviation (*** *p* < 0.001, **** *p* < 0.0001, the significance was analyzed by *T* tests using GraphPad Prism 7 software).

Next, we tested the effects of Mg^2+^, Zn^2+^, NaCl, EDTA, pH, temperature, 5% BSA, and human serum on the antibacterial activity of ClyC. Results showed that Mg^2+^ (Figure 3A), Zn^2+^ (Figure 3B), NaCl (Figure 3C), and EDTA (Figure 3D) inhibited the antibacterial activity of ClyC in a dose-dependent manner. Specifically, 50 μM of Zn^2+^ or EDTA completely abolished the lytic activity of ClyC. High activity was observed at pH ranging from 4 to 10 (Figure 3E) and temperatures below 45 °C (Figure 3F). Surprisingly, enhanced staphylolytic activity of ClyC was observed in PBS containing 5% BSA (Figure 3G) and in PBS containing 0.25–12% human serum (Figure 3H).

### 2.4. Effects of Calcium on the Enzymatic Activity of ClyC

**Figure 4:**
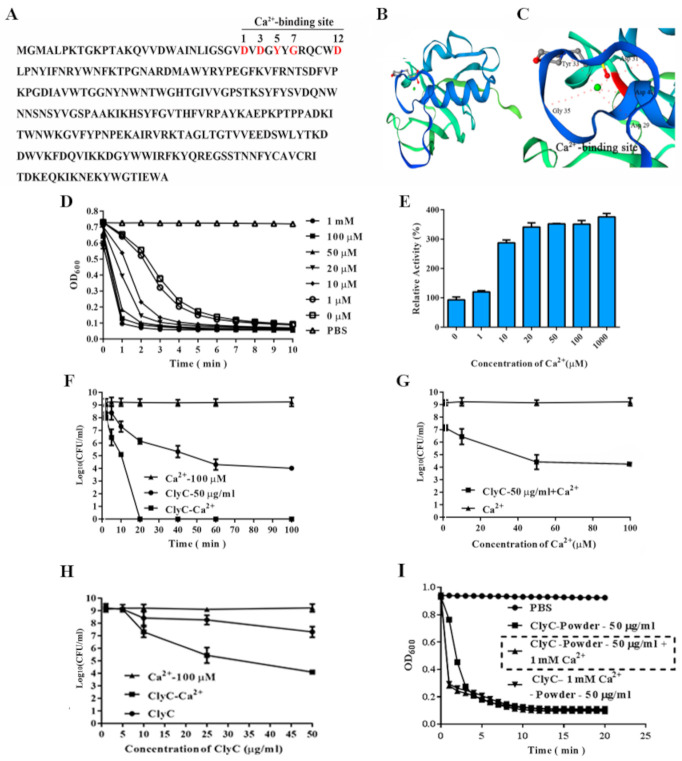
Effects of calcium on the enzymatic activity of ClyC. (**A**) Predicted conserved calcium-binding site (marked red) of ClyC. (**B**) 3D structure of ClyC predicted by SWISS MODEL. (**C**) Molecular docking of calcium into the binding pocket of ClyC. (**D**) Bacteriolytic curves of ClyC (25 μg/mL) against *S. aureus* T23 with different concentrations of calcium. (**E**) Relative activities of ClyC (25 μg/mL) against *S. aureus* T23 at different concentrations of calcium for 1 min. (**F**) Time-dependent survival rate of *S. aureus* T23 after treatment with 100 μM Ca^2+^, 50 μg/mL of ClyC, and 50 μg/mL of ClyC in the presence of 100 μM Ca^2+^. (**G**) Survival rate of *S. aureus* T23 after treatment with 50 μg/mL of ClyC for 10 min in the presence of different concentrations of calcium. (**H**) CFU counts after different concentrations of ClyC were incubated with tested bacteria containing 100 μM Ca^2+^ for 10 min. (**I**) Activity of lyophilized ClyC against *S. aureus* T23 under different conditions. ClyC was lyophilized in the presence/absence of 1 mM CaCl_2_. After re-dissolving the lyophilized powder in ultra-pure water, the lytic activity of ClyC powder lyophilized in the absence of Ca^2+^ was determined in PBS with (ClyC-Powder—50 μg/mL + 1 mM Ca^2+^) and without 1 mM Ca^2+^ (ClyC-Powder—50 μg/mL), the lytic activity of ClyC powder lyophilized in the presence of Ca^2+^ (ClyC- 1 mM Ca^2+^-Powder—50 μg/mL) was determined in PBS, and groups treated with PBS containing 1 mM Ca^2+^ (PBS) were used as controls. All experiments were repeated three times. Error bars represent standard deviation.

Amino acid sequence revealed that there is a conserved Ca^2+^ binding site (D29, D31, Y33, G35, D40, relative position of amino acids: 1, 3, 5, 7, 12) of amino acids in the CHAP domain of Ply187 in the newly formed ClyC (Figure 4A–C). As expected, calcium promotes the activity of ClyC in a dose-dependent manner (Figure 4D–G). Specifically, a reduction of 9 log_10_ (CFU/mL) in viable bacterial number was observed after treating overnight cultured *S. aureus* T23 with 50 μg/mL of ClyC containing 100 μM Ca^2+^ at 37 °C for 20 min (Figure 4F). For Figure 4D,E,G, 100 μM Ca^2+^ still maintains good activity against *S. aureus.* Within 10 min, the concentration of ClyC was about 5× that of ClyC+100 μM Ca^2+^ in order to achieve a similar log reduction (Figure 4H). In addition, ClyC retained a full antibacterial activity after being lyophilized without stabilizing excipients (Figure 4I).

### 2.5. Effects of ClyC Alone against Planktonic and Sessile S. aureus or in Combination with Penicillin G

*S. aureus* strain T23 was used to test the biofilm removal efficacy of ClyC and the combined antimicrobial effects of ClyC and penicillin G. Crystal violet staining assay showed that the biofilm of T23 was removed by ClyC in a dose-dependent manner. Specifically, biofilm could rarely be observed in wells treated with 10 μg/mL of ClyC for 1 h (Figure S2A). In a checkerboard assay, an obvious additive effect was observed between ClyC and penicillin G (Figure S2B). The MIC values of ClyC and penicillin G were 12 and 0.6 μg/mL, respectively. *S. aureus* treated with combinations of two antimicrobial agents (1 μg/mL ClyC and 0.3 μg/mL penicillin G or 6 μg/mL ClyC and 0.2 μg/mL penicillin G) showed ƩFIC of 0.42, which is <0.5 and thus indicates the synergism between the two antimicrobials [29].

### 2.6. ClyC Cytotoxicity In Vitro

The CHO-K1 cell line was used to detect ClyC’s cytotoxicity. As shown in Figure S3A, rare cytotoxicity was observed under a high ClyC concentration of up to 125 μg/mL. This meant that the lower doses of ClyC (<50 μg/mL) used in previous in vivo and in vitro experiments were safe. To further confirm the safety of ClyC administrated in vivo, we measured the expression levels of cytokine IL-8 in mice serum after administration of 0.1 mg/mouse of ClyC (*n* = 5) at different times post-infection. As shown in Figure S3B, compared with the PBS-treated group, no significant difference in the expression levels of IL-8 was observed in the ClyC-treated group at 2, 3, 5, and 7 h post infection (h.p.i).

### 2.7. ClyC Protects Mice from Lethal S. aureus Infection

**Figure 5:**
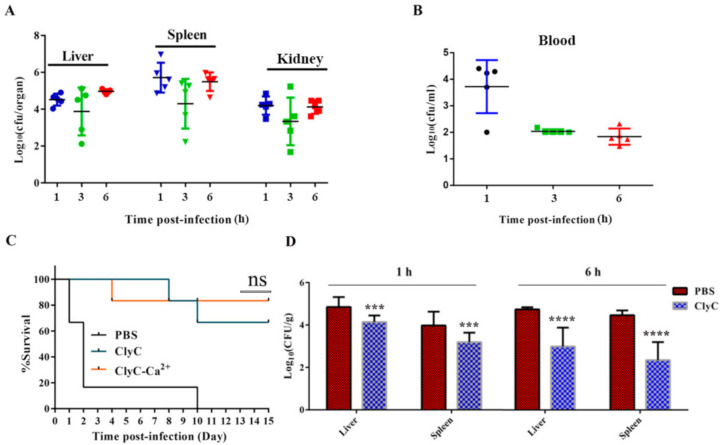
ClyC protects mice from lethal *S. aureus* infection. Bacterial burden in organs (**A**) and blood (**B**) at 1, 3, and 6 h.p.i with a lethal dose of MRSA T23. (**C**) Protective efficacy of ClyC. Female BALB/c mice were infected with a lethal dose of *S. aureus* T23 and divided into three groups (*n* = 6) randomly. The groups were intraperitoneally injected with either 0.1 mg/mouse of ClyC, 0.1 mg/mouse of ClyC containing 100 μM Ca^2+^, or an equal volume of PBS at 3 h.p.i, respectively. Survival rates in all groups were recorded for 15 days. (**D**) Bacterial burden in mice liver and spleen after treatment with ClyC or PBS for 1 h and 6 h. Female BALB/c mice were infected with a lethal dose of *S. aureus* and randomly divided into four groups (*n* = 5). Each group was then intraperitoneally injected with 0.1 mg/mouse of ClyC 1 h.p.i, organs collected at indicated time points, and bacterial burden in liver and spleen were counted after homogenization by a QIAGEN TissueLyse II homogenizer. (*n* = 5 *** *p* < 0.001, **** *p* < 0.0001, the significance was analyzed by *T* tests using GraphPad Prism 7 software).

A mouse systemic infection model was established to determine the protective efficacy of ClyC in vivo. Female BALB/c mice were challenged interperitoneally with a lethal dose of 3 × 10^8^ CFU/mouse of *S. aureus* T23. Large bacterial loads could be detected in the liver, spleen, kidney (Figure 5A), and blood (Figure 5B) of the infected mice at 1 h.p.i. Subsequently, mice received a single dose of 0.1 mg/mouse of ClyC, 0.1 mg/mouse of ClyC containing 100 μM Ca^2+^, or an equal volume of PBS at 1 h.p.i with a lethal dose of *S. aureus* T23. As shown in Figure 5C, the survival rates were 66%, 83%, and 0% in groups treated with 0.1 mg/mouse of ClyC, 0.1 mg/mouse of ClyC containing 100 μM Ca^2+^, and PBS, respectively. To further understand the mechanism of protection of ClyC, we determined the bacterial burden in liver and spleen of the infected mice after treatment with ClyC or PBS at 1 and 6 h.p.i. Results showed that bacterial loads decreased significantly in organs from the ClyC-treated group compared to those of the PBS-treated group. In particular, a reduction of 2 Log_10_ (CFU/g) was observed in the group that received ClyC treatment at 6 h.p.i (Figure 5D).

## 3. Discussion

Lysins are considered as novel alternatives against multidrug-resistant pathogens [7,30]. Chimeric lysins, which are derived from natural lysins, have been shown to have improved properties compared to natural lysins [31,32]. In this study, a novel chimeric lysin, ClyC, was constructed by fusing the CD from the Ply187 lysin with the CBD from the LysSA97 lysin. The CD of Ply187 (1–157aa, Ply187N) was chosen because it has been shown to have much higher activity compared to its parental full length *S. aureus* phage Ply187 [25]. Similar to other studies [15,26,33,34], this activity can be further improved by binding it to the CBD of another lysin to form a novel chimeric lysin. The non-SH3b CBD of LysSA97 has a specific binding spectrum to staphylococcus cells [28], hence used to form the new chimera, ClyC. The chimera was found active against all MRSA strains and other staphylococcal species tested, with a wide pH range from 4 to 10 (retained a high lytic activity above 30% of the maximum), making it different from most other lysins which regularly shows good activity in a pH range of 5~8 [35,36]. This wide tolerance to pH has also been reported on the CHAP domain of Ply187 used in this study [37]. Unlike some proteins which are inactive in the presence of serum or bovine serum albumin (BSA) [38], a higher activity was observed in ClyC in the presence of 5% BSA.

*S. aureus* are capable of causing biofilms that can make them less susceptible to antibiotics compared to planktonic bacteria [39,40]. Here, ClyC could remove *S. aureus* biofilms in a dose dependent manner. Specifically, after 1-h exposure to a dosage of 10 μg/mL of ClyC, biofilms formed by MRSA T23 could rarely be observed. This means that ClyC has the potential to be used for topical removal of *S. aureus* biofilms and treatment of *S. aureus* biofilm-related infections as suggested in previous studies [31,40]. Additionally, lysins have also been suggested to have synergistic interactions with antibiotics [41,42]. A staphylococcal specific lysin, ClyS, showed synergistic effects in vitro (ΣFIC < 0.5) with two commonly used antibiotics (oxacillin and vancomycin) to treat *S. aureus* infections [42]. Similarly, ClyC had a synergistic effect with penicillin G in vitro (ΣFIC < 0.5). Penicillin G is a broad-spectrum antibiotic capable of inhibiting a wide range of bacterial infection including staphylococcal infections. In this study, we speculate that the in vivo synergy between penicillin G and ClyC is due to the enhanced lysis of the *S. aureus* bacteria.

Besides its high activity in normal buffers, ClyC showed significantly enhanced activity in the presence of Ca^2+^. Looking at the amino acid sequence of ClyC, there is one conserved calcium binding site in its catalytic domain (relative position 1, 3, 5, 7, 12) and four aspartic acids in its C terminus (D residues in locations 193, 200, 201, 206 of ClyC). It has been reported that three aspartic acid residues can bind Ca^2+^ [24]. Therefore, ClyC may possess two calcium-binding sites, which may be the reason why ClyC’s activity is highly enhanced by Ca^2+^. Additionally, using a similar CHAP domain of Ply187, Meng et.al found that different ions including calcium ions could increase the activity of Ply187 [37]. In order to achieve a similar effect, the concentration of ClyC was about 5× that of ClyC+Ca^2+^. However, this experiment was done within 10 min and we do believe that the activity of ClyC can increase with an increase in time beyond 10 min. Although several lysins [24,36,43] have shown Ca^2+^ dependent activity, this is the first time to see that Ca^2+^ could enhance the activity of ClyC dramatically, with up to 9 Log_10_ (CFU/mL) *S. aureus* cell reduction within 20 min in the presence of Ca^2+^. As far as we know, most lysins against *S. aureus* have a log reduction of between 2–4 logs and only PlySs2 lysin [18] (described as one of the broadest acting lysins) has shown a high reduction of about 5 logs against MRSA which is still lesser than that of ClyC.

The activity of ClyC was increased in the presence of human serum. This was mainly because human serum contains large amounts of free calcium [22]. The calcium-enhanced bactericidal activity of ClyC would mean that less protein needs to be used in treating sepsis since there naturally exists Ca^2+^ in human blood. We also believe that the higher antibacterial activity observed in 5% BSA was possibly due to the residual calcium in BSA, which is usually produced from bovine blood.

Lyophilization is a widely used method to keep proteins stable during storage [44]. Excipients such as BSA and sugars are regularly needed to prevent proteins from denaturation during freeze-drying. ClyC was found able to maintain good antibacterial activity without any stabilizing excipients. This unique property could mean that ClyC may be a robust lysin for further developments.

The cytotoxicity assay and mouse model tests demonstrated that ClyC has low cytotoxicity and good protective efficacy at a low dose of 0.1 mg/mouse. In comparison, a higher dose was needed for other lysins to achieve comparable protection. For example, ClyH was reported to protect 80% of lethal *S. aureus* challenged mice under a higher dose of 1 mg/mouse [33]. Using a small dose of lysin for treatment would not only ensure safety because of its dose-dependent cytotoxicity in human cell lines [14], but also benefit the reduction of immune responses which might lead to side effects. Despite the in vitro increase in bactericidal activity of ClyC in the presence of Ca^2+^, there was no significant difference between the survival rate of the mice treated with ClyC and those treated with ClyC plus 100 μM Ca^2+^. The reason was speculated to be due to the natural existence of calcium ions in the mice body which could have improved the activity of ClyC and masked the effects of the additional Ca^2+^.

## 4. Materials and Methods

### 4.1. Ethical Consideration

All mouse experiments were carried out in an ABSL-2 lab. The experimental protocols were carried out following the regulations and guidelines set forth by the Animal Experiments Committee of Wuhan Institute of Virology, Chinese Academy of Sciences, and approved by the committee (No: WIVA17201602).

### 4.2. Bacterial Strains

All bacterial expression strains used in this study are listed in Table S1. *S. aureus* strains including Methicillin-resistant *S. aureus* T23 were cultured in lysogeny broth (LB) medium at 37 °C. *Streptococcus agalactiae* were grown in brain heart infusion (BHI) broth at 37 °C. *Escherichia coli* BL21 (DE3), used for gene cloning and protein expression, was grown in LB medium, and 50 μg/mL of kanamycin was supplemented when needed (Supplementary Materials Table S1). Bacterial loads in mice organs (liver, kidney, and spleen) and blood were counted in Baird-Parker agar plates containing 5% egg-yolk tellurite emulsion.

### 4.3. Construction of Expression Plasmids and Protein Purification

The ClyC to be constructed in this study consisted of the CD of Ply187 (Ply187CD, amino acids 1–157, GenBank: CAA69022.1) [31] and the CBD of LysSA97 (SA97CBD, amino acids 370–470, GenBank: AHZ95694.1) [32]. Using the four primers A (Ply187CD-F), B (Ply187CD-R), C (SA97CBD-F), and D (SA97CBD-R) listed in Table S2, ClyC was constructed by using a PCR-driven overlap extension technique. Firstly, *ply187CD* gene and *SA97CBD* gene were obtained by two independent PCRs using A+B and C+D as primers and the plasmids pET28a-Ply187CD and pET28a-SA97CBD (synthesized by Sangon Biotech, Shanghai, China) as templates, respectively. Resultant PCR products and the primers A and D were used for a third PCR to amplify the *ClyC* gene. Afterwards, the PCR product was cloned into the *Nco*I and *Xho*I sites of the pET28a (+) plasmid (GenBank: MK847907.1). Finally, the recombinant plasmid pET28a-ClyC was transformed into *E. coli* BL21 (DE3). The sequence of the chimeric ClyC gene was confirmed by sequencing.

The expression strain of *E. coli* BL21 cells were incubated in LB medium containing 0.1 mg/mL of kanamycin to an OD_600_ of 0.4~0.6, then induced with 0.2 mM isopropyl β-D-thiogalactoside (IPTG) at 16 °C for 18 h to allow protein expression. Cells were then harvested by centrifugation at 8000× *g* for 5 min, resuspended in 20 mM imidazole, lysed by a cell disrupter on ice, and finally centrifuged at 10,000× *g* for 30 min to remove cell debris. His-tagged proteins were purified by affinity chromatography using nickel nitrilotriacetic acid columns. Target proteins were collected by washing and eluting with 40- and 250-mM imidazole, respectively. Collected proteins were dialyzed against PBS buffer (137 mM NaCl, 2.7 mM KCl, 4.3 mM Na_2_HPO_4_·H_2_O, 1.4 mM KH_2_PO_4_, pH 7.4). The dialyzed protein was then passed through a Detoxi-Gel™ Endotoxin Removing Gel (Thermo Scientific, Waltham, MA, USA) and quantified by a ToxinSensor Chromogenic LAL Endotoxin Assay Kit (GenScript, Nanjing, China).

### 4.4. Lytic Activity Assay

The bacteriolytic activity of ClyC was determined by the turbidity reduction assay. Briefly, cells of bacterial strains to be tested were harvested by centrifugation at 10,000× *g* for 1 min, resuspended in PBS buffer (pH 7.4), and mixed with ClyC (0, 25, 50, and 100 μg/mL) to a final OD_600_ of 0.8~1.2. The decrease of samples in OD_600_ was monitored by a Synergy H1 microplate reader every 30 s for 10 min at 37 °C.

To test the lytic spectrum of ClyC, multiple strains of streptococci (including *S. pyogenes*, *S. agalactiae*, and *S. pneumoniae*), staphylococci (including *S. aureus*, *S. epidermidis*), and listeria were tested for sensitivity to ClyC. Overnight cultured strains were washed once with PBS and resuspended to a final OD_600_ of 0.8~0.9. The cultures were then treated with 25 μg/mL of ClyC at 37 °C for 10 min, and the OD_600_ of each treatment was monitored by a microplate reader. Susceptibility to ClyC was defined as the net change in OD_600_ yielded from the final OD_600_ subtracted from that of the PBS treated control well. For the plaque assay, 0.02–1.65 μg of ClyC in a volume of 5 μL was spotted onto agar plates overlaid with *S. aureus* and incubated overnight at 37 °C. Bactericidal activity was expressed by clear zones on the agar plates. Viability of treated cells measured as log CFU/mL was determined by serial dilution and plating to LB agar plates.

### 4.5. Lyophilization

ClyC only and ClyC plus CaCl_2_ were lyophilized using the LyoBeta 6PL (Telstar, Spain) instrument. Briefly, 0.5 mL of ClyC (0.5 mg/mL) and ClyC plus CaCl_2,_ in PBS were loaded into a clean 5mL bottle and lyophilized under the following conditions: freezing at −30 °C for 4 h, condenser preparation for 15 min, chamber vacuum at 400 μbar atmosphere pressure, primary drying at −40 °C for 13 h at 200 μbar atmosphere pressure, and a final secondary drying at 4 °C for 6 h. Three days post freeze drying, the lyophilized powders were re-dissolving in ultra-pure water to test for the lytic activity of ClyC.

### 4.6. MIC of ClyC Alone and in Combination with Penicillin G

To determine the minimum inhibitory concentrations (MICs) of ClyC, we tested 26 strains of MRSA and 3 strains of methicillin-sensitive *S. aureus*. Briefly, *S. aureus* strains were inoculated in LB at a final concentration of 1 × 10^5^ cells/well into a 96-well microtiter plate and incubated for 24 h at 37 °C in the presence of different concentrations of ClyC (0, 1, 2, 4.5, 9, 18, and 36 μg/mL). MIC was defined as the lowest concentration of ClyC inhibiting visible growth.

Synergistic antimicrobial action between ClyC and penicillin G against *S. aureus* was tested in vitro using the checkerboard assay. Briefly, *S. aureus* T23 was inoculated at a final concentration of 1 × 10^5^ cells/well in a 96-well microtiter plate, and allowed to incubate in LB for 24 h at 37 °C in the presence of varying concentrations of ClyC (0, 1, 2, 4, 8, 16, 32, and 64 μg/mL), penicillin G (0, 0.25, 0.5, 1, 2, 4, 8, and 16 μg/mL), or a mixture of ClyC and penicillin G. The sum of the fractional inhibitory concentrations (FICs) of both antimicrobials (ƩFIC = FIC_ClyC_ + FIC_penicillin G_ = MIC_ClyC + penicillin G_/MIC_ClyC_ + MIC_penicillin G + ClyC_/MIC_penicillin G_) was calculated.

### 4.7. Effects of Different Factors on ClyC Activity

*S. aureus* T23 cells were suspended in phosphate buffer (PBS, pH 7.4) to a final OD_600_ of 1.0 containing various concentrations of EDTA (ranging from 0 to 800 μM), Ca^2 +^ (CaCl_2_ ranging from 0 to 1000 μM), Mg^2+^ (MgCl_2_ ranging from 0 to 5000 μM), Zn^2+^ (ZnCl_2_ ranging from 0 to 100 μM), NaCl (ranging from 0 to 1000 μM), and human serum (ranging from 0.25 to 12%). The lytic activities of ClyC at different pH values were also measured using a universal buffer as previously described [35]. ClyC was then added to the suspensions to a final concentration of 25 μg/mL. Finally, the OD_600_ of the mixtures was monitored by a microplate reader at 37 °C for 60 min. The residual activity of ClyC (25 μg/mL) against *S. aureus* T23 was measured as the decrease in OD_600_ within 60 min after treatment at different temperatures ranging from 4 to 65 °C for 30 min. *S. aureus* T23 cells were first suspended in PBS containing 5% BSA to a final OD_600_ of 0.8. The cells were then incubated with different concentrations of ClyC (0, 25, 50, 100, and 200 μg/mL) at 37 °C for 60 min. The mixtures were finally serially diluted and plated onto LB agar for CFU count of live *S. aureus*.

### 4.8. Construction of 3D Models of ClyC

Amino acid sequences of the CD of Ply187 (GenBank: CAA69022.1) [26] and the CBD of LysSA97 (GenBank: AHZ95694.1) [28] were downloaded from the National Center for Biotechnology Information (NCBI) database. The 3D protein model structures were constructed to analyze their Ca^2+^ binding sites using the SWISS-MODEL server [45].

### 4.9. ClyC Cytotoxicity Assay

The cytotoxicity of ClyC was studied using MTT (3-(4, 5-dimethylthiazolyl-2)-2, 5-diphenyltetrazolium bromide) assay. Briefly, 100 µL of CHO-K1 cells (Chinese hamster ovary cells) were seeded into a 96-well plate at a final concentration of 5 × 10^3^ cells/well and allowed to adhere to the surface for 24 h at 37 °C under 5% CO_2_. Next, the medium was removed, and new medium containing different concentrations of filter-sterilized ClyC (0, 15.625, 31.25, 62.5, 125, 250, and 500 μg/mL) was added to each well and allowed to co-culture for 24 h at 37 °C under 5% CO_2_. Afterwards, 10 μL of MTT (5 mg/mL) was added to each well and incubated for 4 h at 37 °C under 5% CO_2_. MTT was then removed, and 200 μL of DMSO was added into each well and mixed for 10 min at a speed of 100 rpm/min. The OD_570_ of each well was finally read by a microplate reader. The medium without ClyC served as the positive control (PC), while medium without ClyC and CHO-K1 cells served as the negative control (NC). The relative cytotoxicity of cells was calculated as: %cytotoxicity = [1 − (OD_sample_ − OD_NC_)/(OD_PC_ − OD_NC_)] × 100%.

#### 4.9.1. Biofilm Removal Efficacy of ClyC

Crystal violet (CV) staining assay was used to determine the biofilm removing capability of ClyC. Briefly, 10 µL of overnight cultures of *S. aureus* T23 and 190 µL of TSBG (TSB + 1% glucose) were dispensed into wells of a 96-well polystyrene plate (Tissue culture treated, Nest, China), and incubated at 37 °C for 24 h to allow biofilm formation. After removing the supernatant and washing three times with sterile PBS, biofilms were treated with 200 µL of different concentration of ClyC or PBS (control) for 1 h. After washing twice with PBS, wells were dried and stained with 100 μL of 4% CV/well for 5 min at 37 °C. Following that, wells were washed with sterile PBS twice carefully, and resuspended in 200 µL of absolute ethanol. Finally, OD_600_ of each well was read by a microplate reader. The biofilm removal efficacy was presented as the difference in OD_600_ between ClyC and PBS treated wells.

#### 4.9.2. Mouse Infection Model

To test the protective efficacy of ClyC, female BALB/c mice, 6–8 weeks old, were injected intraperitoneally with a lethal dose of *S. aureus* T23 and divided into three groups (6 each). Three hours post infection (h.p.i), groups were administrated intraperitoneally with either 0.1 mg/mouse of ClyC, 0.1 mg/mouse of ClyC containing 100 μM Ca^2+^, or an equal volume of PBS. The survival rates for all groups were recorded for 15 days. Meanwhile, bacterial loads in blood and organs were also tested at different times (1, 3, and 6 h.p.i). Briefly, blood was collected and serially diluted for CFU count on LB agar. Kidney, liver, and lungs were aseptically removed from each mouse, infiltrated in 1 mL PBS with 0.1% Triton X-100, and prepared to tissue homogenates using a QIAGEN Tissue Lyse II homogenizer at 1/20 frequency for 90 s. Finally, serial dilutions of each organ were plated on Baird-Parker agar for CFU count. Interleukin 8 (IL-8) level in blood was also measured using a commercial ELISA kit (Shengdawei technology, China). Here, sera were separated from the mice blood by centrifugation at 3000 *g* for 30 min, and IL8 was detected following the instructions of the kit.

## 5. Conclusions

In conclusion, we report here a novel chimeric lysin, ClyC, that possess robust and stable bactericidal activity against *S. aureus* in vitro and in vivo without foreseeable harmful effects. Its Ca^2+^ enhanced activity means that a much smaller amount of ClyC could be used in vivo, which would reduce possible side effects and improve its therapeutic index.

## Data Availability

The data presented in this study are available on request from the corresponding author.

## Supplementary Materials

The following are available online at https://www.mdpi.com/article/10.3390/antibiotics10040461/s1, Figure S1: Lytic spectrum of ClyC, Figure S2: Effects of ClyC alone against planktonic and sessile *S. aureus* or in combination with penicillin G, Figure S3: Cytotoxicity of ClyC, Table S1: Bacterial strains used in this study, Table S2: Primers used in the overlap PCR, Table S3: MIC of ClyC against different *S. aureus* strains.
