# Supplementary material for: A Highly Active Chimeric Lysin with a Calcium-Enhanced Bactericidal Activity against Staphylococcus aureus In Vitro and In Vivo
Source: Antibiotics (Basel). 2021 Apr 19;10(4):461. doi: 10.3390/antibiotics10040461 (PMC8074178; doi:10.3390/antibiotics10040461)
Supplement: Supplementary file 1 [file antibiotics-10-00461-s001.zip › antibiotics-1174246-supple.pdf]

antibiotics

MDPI

Supplementary Information

A highly active chimeric lysin with a calcium-enhanced bactericidal activity against Staphylococcus aureus in vitro and in vivo

Xiaohong Li [1,2§], Shujuan Wang [1,2§], Raphael Nyaruaba [1,2], Huan Liu [1,2], Hang Yang [1,2*], and Hongping Wei [1,2*]

Table S1. Bacterial strains used in this study.

Strain	Description
BL21(DE3)	F⁻ *ompT hsdS*$_B$ (r_B^- m_B^-) *gal dcm* λDE3 (harboring gene *1* of the RNA polymerase from the phage T7 under the *PlacUV5* promoter)
E. coli BL21/pET28a-ClyC	Constructed in this study
E. coli BL21/pET28a-Ply187CD	pET28a derivative carrying the catalytic domain of Ply187 gene
E. coli BL21/pET28a-SA97CBD	pET28a derivative containing the cell-wall binding domain of LysSA97 gene

Table S2: Primers used in the overlap PCR.

Code	Name	Sequence
A	Ply187CD-F	ATATCCATGGGCATGGCACTGCCTAAAACGGG
B	Ply187CD-R	AGTTCCAGGTGATCTTGTCCGCTGGTGGTG-TAGGTTTCGGTTC
C	SA97CBD-F	GAACCGAAACCTACACCACCAGCGGACAAGATACCTG-GAACT
D	SA97CBD-R	TATACTCGAGCGCCCATTCGATGGTGCCCCAG

Table S3. MIC of ClyC against different *S. aureus* strains.

S. aureus strains	Drug resistance	MIC of ClyC (μg/mL)
WHS11016	Methicillin-resistant	18
WHS11095	Methicillin-resistant	18
WHS11048	Methicillin-resistant	18
WHS11101	Methicillin-resistant	18
WHS11044	Methicillin-resistant	18
WHS11051	Methicillin-resistant	18
WHS11036	Methicillin-resistant	9
WHS11098	Methicillin-resistant	18
WHS11034	Methicillin-resistant	18
WHS11009	Methicillin-resistant	9
WHS11040	Methicillin-resistant	9
AB91118	Methicillin-sensitive	9

N315	Methicillin-resistant	18
WHS11024	Methicillin- sensitive	4.5
WHS11041	Methicillin-resistant	18
WHS11011	Methicillin-resistant	9
WHS11018	Methicillin-resistant	18
WHS11032	Methicillin-resistant	18
WHS11005	Methicillin-resistant	18
WHS11099	Methicillin-resistant	9
WHS11046	Methicillin-resistant	36
WHS11100	Methicillin-resistant	18
WHS11026	Methicillin-resistant	18
WHS11037	Methicillin-resistant	9
WHS11033	Methicillin-resistant	18
WHS11025	Methicillin-resistant	18
ATCC29213	Methicillin- sensitive	18
WHS11102	Methicillin-resistant	36
WHS11017	Methicillin-resistant	18

Figure S1. Lytic spectrum of ClyC.

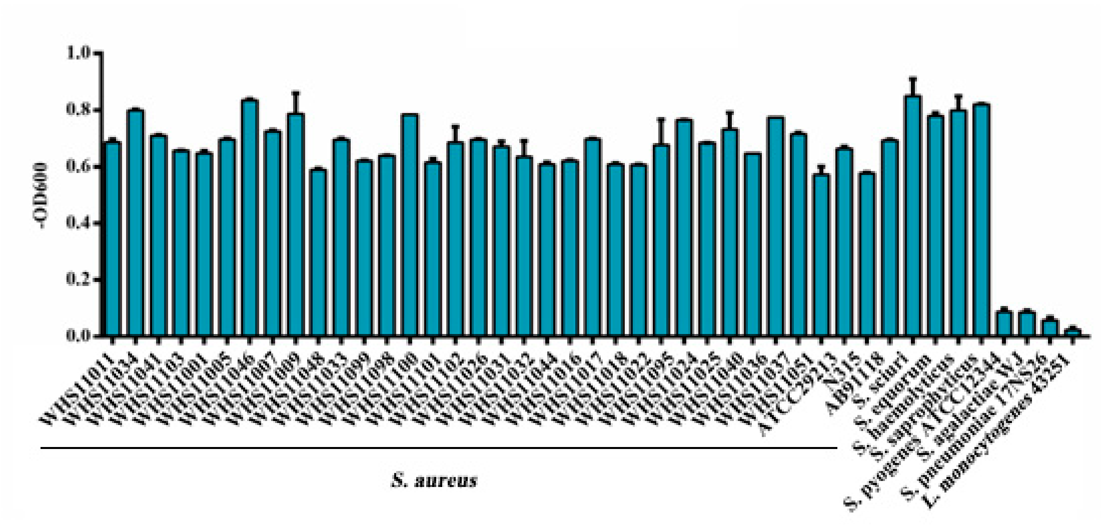

Fig S1. Susceptibility of ClyC to staphylococcus and single strains of *S. pyogenes, S. agalactiae, S. pneumoniae,* and *Listeria monocytogenes*. Strains were washed once with PBS and resuspended to a final OD_{600} of 0.8～0.9. After treatment with 25 μg/mL of ClyC at 37 °C for 10 min, the final OD_{600} value of treated wells were subtracted from the PBS-treated well to yield the net change in OD_{600}, and the bactericidal efficiency determined by the value change of OD_{600}. The experiment was repeated three times. Error bars represent the standard deviations.

Figure S2. Cytotoxicity of ClyC.

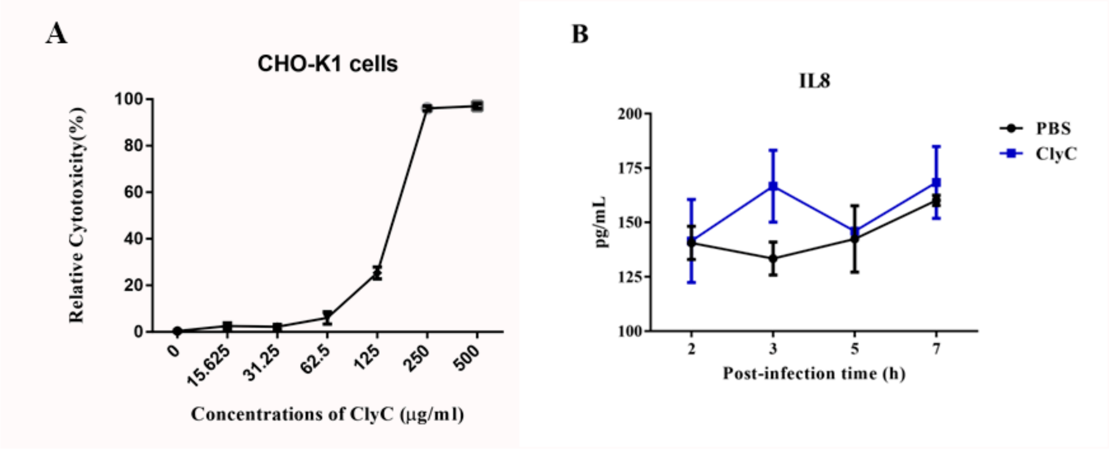

Fig S2. Cytotoxicity of ClyC. (A) Cytotoxicity of ClyC to CHO-K1 cells. Cells were co-cultured with different concentrations of ClyC (0, 15.625, 31.25, 62.5, 125, 250, and 500 μg/mL) for 24 h and the relative viability was calculated by the change of OD_{570} by MTT assay. (B) Concentration of IL-8 in mice serum at different times post ClyC treatment.
